# The Quick and Dirty: A Tetanus Case Report

**DOI:** 10.5811/cpcem.2019.1.41301

**Published:** 2019-01-22

**Authors:** Patrick Mcelaney, Masayuki Iyanaga, Stormy Monks, Edward Michelson

**Affiliations:** Texas Tech University Health Sciences Center El Paso, Department of Emergency Medicine, El Paso, Texas

## Abstract

Tetanus is an increasingly rare diagnosis in the post-vaccination era, although it continues to have significant morbidity and mortality worldwide. In the United States (U.S.), the incidence of tetanus has declined dramatically due to the widespread use of the vaccine. High-risk populations for tetanus in the U.S. include the elderly, diabetics, injection drug users, and unvaccinated individuals. This is a report of a 78-year-old male with an incomplete immunization history who presented to an emergency department with jaw pain and who was ultimately diagnosed with tetanus. This report highlights the importance of prompt diagnosis, treatment, and prevention of tetanus.

## INTRODUCTION

Tetanus is a life-threatening disease caused by a toxin produced by *Clostridium tetani*, an anaerobic gram-positive, spore-forming bacillus found in soil and animal excrement.^1^ Tetanus is an acute illness diagnosed clinically with features of hypertonia and muscle spasm in the absence of a more likely diagnosis.^2^ Tetanus occurs when bacterial spores enter the body through breaks in the skin and then germinate under anaerobic conditions. The bacteria produce an exotoxin – tetanospasmin – which binds at the neuromuscular junction and results in painful muscle contractions.^2^

There are three different clinical forms in which tetanus can be classified: generalized; localized; and cephalic.^3^ Generalized tetanus is the most common presentation with patients initially experiencing spasms of the masseter muscle or trismus. This progresses to painful, generalized spasms of muscles in the neck, abdomen, or extremities and potential abdominal rigidity.^3^ Patients may also develop autonomic instability, which may contribute to cardiac arrest. Localized tetanus occurs with spasm to a certain muscle group, typically near the wound.^3^ Cephalic tetanus, the most rare form, is associated with wounds to the head or face. It presents differently from the other forms with cranial nerve palsies resulting in flaccid paralysis rather than spasm. Both localized and cephalic tetanus can progress to the generalized form.^3^ Upon recognition of tetanus, prompt treatment is recommended with tetanus immunoglobulin, tetanus toxoid, aggressive wound care, and antibiotics with coverage for anaerobes. Medications for treatment of muscle spasm should be used, with benzodiazepines being the preferred agent.^3^

Since 1947 when the disease was first tracked by the Centers for Disease Control and Prevention (CDC), the number of tetanus cases has declined by more than 95% and deaths have declined by greater than 99%.^1^ Only 29 cases of tetanus were reported in the United States (U.S.) in 2015, yet it continues to be a morbid disease, with a case-fatality rate of 13.2%.^1^ Populations at an increased risk for infection include unvaccinated individuals, the elderly, diabetics and illicit-injection drug users.^1^ Knowing how to identify and treat at-risk wounds and cases of suspected tetanus is essential for emergency physicians.

## CASE REPORT

A 78-year-old Hispanic male, a resident of Mexico, presented to the emergency department (ED) of a level I county trauma center with a complaint of jaw pain for the prior three days. On review of systems, the patient also complained of abdominal bloating. His medical history was only significant for hypertension, although he did not take any medications. The patient did not report having allergies, and he denied the use of tobacco, alcohol, or drugs. His vital signs were as follows: temperature 36.9**°** Celsius, blood pressure 165/109 millimeters of mercury, pulse 88 beats per minute, and respiratory rate 18 breaths per minute.

The patient appeared to have difficulty opening his mouth and exhibited dysphonia as a result. He had no reproducible pain on exam, but was uncomfortable when we attempted to open his mouth by force. While the oral exam was limited secondary to poor mouth opening, no caries or abscesses were appreciated. No lymph nodes were palpable and the remainder of the ear, nose, and throat exam was unremarkable. The patient’s abdomen was rigid and mildly distended, but non-tender. On examination, the medial aspect of the right forearm revealed a healing laceration, approximately 5 × 2 centimeters. When questioned about the wound, the patient stated he had received it at work two weeks prior when he fell off a tractor and into muddy water. He stated he had been seen by a doctor in Mexico for the wound and was given a topical medication, which he had been applying. When asked about immunization status, the patient denied receiving tetanus prophylaxis for the wound and stated that as far as he could remember he had never received any childhood or adult vaccinations.

The patient’s blood tests and computed tomography of the head and neck were within normal limits. Based on exam and history, the likely diagnosis of tetanus was made. His wound was debrided. Based on CDC guidelines, we administered tetanus immunoglobulin, tetanus diphtheria and pertussis vaccine, and intravenous metronidazole. The patient was admitted to the medical intensive care unit (MICU) for further treatment.

In the MICU, the patient was treated symptomatically with two milligrams of lorazepam as needed for muscle spasms. On hospital day four, he had an apneic event and subsequent cardiac arrest. He was intubated with return of spontaneous circulation. He was unable to be weaned from the ventilator due to continued trismus. On hospital day 11, the patient had a tracheostomy and percutaneous gastrostomy tube placed, given concern for prolonged course on the ventilator. He had continued improvement in mental status along with fewer episodes of tetany. On hospital day 16, the tracheostomy became dislodged without respiratory compromise, and so was discontinued. On hospital day 18, the patient was transferred to the general medical ward. He was started on a clear liquid diet and was able to advance to full liquids during his stay. He was discharged to home on hospital day 22 with the formal diagnosis of tetanus.

## DISCUSSION

Tetanus is a clinical diagnosis defined by hypertonia with painful muscle contractions or spasm. Generalized tetanus is a disease characterized as a progression from trismus to stiffness of neck muscles with difficulty swallowing. Patients may develop generalized muscle spasms including spasms of the abdominal musculature that can present as a rigid abdomen.^2^ The differential diagnosis for those presenting with trismus and muscle spasm as seen in tetanus includes dystonia, strychnine poisoning, dental infection, seizure, or hypocalcemic tetany.^4^ Patients with tetanus may also initially present with non-specific symptoms including weakness, dysphagia, facial pain, and trismus.

**CPC-EM Capsule**

What do we already know about this clinical entity?

*Tetanus is a well-known life threatening disease*.

What makes this presentation of disease reportable?

*This case reports on a classic presentation on what is becoming an increasingly rare disease since widespread use of the vaccine*.

What is the major learning point?

*Certain groups remain high risk for tetanus, especially when unvaccinated or under-vaccinated*.

How might this improve emergency medicine practice?

*Readers will understand the presenting features, the treatment, and prevention of tetanus*.

Laboratory tests and imaging may aid in evaluating for other diagnoses, but do not assist in confirmation of tetanus.^1^ Given that the diagnosis of tetanus is purely clinical and patients commonly present with non-specific features, the diagnosis on initial presentation may be difficult. However, patients with tetanus should all be admitted for monitoring.^3^ Multiple case reports have described patients who presented with tetanus-like symptoms, but were initially diagnosed and treated for otitis media or sinusitis.^5^,^6^ In patients who present with symptoms associated with tetanus, obtaining a vaccination history and a history of recent wounds is warranted in order to risk stratify.

Prior to the advent and implementation of a widespread vaccination program in the 1940s there were an average of 500–600 cases per year in the U.S. Since then, the incidence of tetanus has greatly decreased. During the most recent CDC report from 2001–2008 there was a total of 233 cases during that eight-year time period.^1^ Despite the decline in cases, there remains a significant population who are at increased risk of the disease. According to the CDC, populations at increased risk include individuals over 65 years old, diabetics, and illicit-injection drug abusers.^1^ In addition, foreign-born individuals have an increased incidence.^1^ This disparity is likely secondary to decreased vaccination rates among minorities and foreign-born individuals.^7^–^9^ The increasing numbers of immigrants to the U.S. from countries with lower immunization rates may result in more cases of tetanus presenting in coming years.^10^

While a diagnosis of tetanus is rare, the treatment of wounds that are at risk of having tetanus contamination remains a common occurrence in EDs in the U.S. In a prospective observational study at five university-affiliated hospitals, researchers found that emergency physicians greatly undertreat at-risk patients who may have inadequate primary immunizations. Specifically, this study reported that 504 patients were identified as having inadequate primary immunizations, and none of the patients received appropriate tetanus prophylaxis.^11^

The CDC recommends tetanus vaccine alone for patients who have clean, minor wounds and unknown or fewer than the three-shot series. However, if the patient presents with a wound that is neither clean nor minor, tetanus immunoglobulin should be administered in addition to the tetanus vaccine (Table).^12^ Furthermore, major and/or dirty wounds require aggressive local wound care and possibly antibiotics. Major wounds include avulsions, punctures, burns, and/or crush injuries. Dirty wounds include those contaminated with dirt, soil, feces, or saliva.^2^ Those with up-to-date tetanus vaccination do not require vaccination for clean or dirty wounds.^12^

## CONCLUSION

Many patients presenting to the ED for wound care will be unimmunized for tetanus or under-immunized with non-protective antibody titers. At-risk patients include individuals over 50 years old and immigrants who may not have been fully immunized.^13^,^14^ As pointed out by Sanchez-Gonzalez et al., the latter group includes “all foreign-born, irrespective of their birthplace, citizenship, language and years of residence in the United States.” 8 According to the 2010 U.S. census, 12.9% or 40 million people in the U.S. are foreign born^7^ and as of 2016, 49.2 million Americans are 65 years or older.^15^ It is imperative to scrutinize the immunization history in these high-risk populations and administer immunoglobulin and tetanus vaccine as recommended. The possibility of tetanus, though rare, should be included in the differential diagnosis of patients from high-risk groups presenting with the typical pattern of muscular rigidity.

## Figures and Tables

**Table:** Centers for Disease Control and Prevention guide to tetanus prophylaxis with tetanus immunoglobin in routine wound management.

History of absorbed tetanus toxoid-containing vaccines (doses)	Clean, minor wound		All other wounds*	
	
	DTaP, Tdap, or Td†	TIG‡	DTaP, Tdap, or Td†	TIG‡
Unknown or <3	Yes	No	Yes	Yes
≥3	No§	No	No¶	No

*DTaP*, diphtheria and tetanus toxoids and acellular pertussis vaccine; *Tdap*, tetanus toxoid, reduced diphtheria toxoid, and acellular pertussis; *Td*, tetanus and diphtheria toxoids; *TIG*, tetanus immune globulin.

* Such as, but not limited to, wounds contaminated with dirt, feces, soil, and saliva; puncture wounds; and wounds resulting from missiles, crushing, burns, and frostbite.

† DTaP is recommended for children <7 years of age. Tdap is preferred to Td for persons aged 11 years or older who have not previously received Tdap. Persons 7 years or older who are not fully immunized against pertussis, tetanus or diphtheria should receive one dose of Tdap for wound management and as part ofg the catch-up series.

‡ People with HIV infection or severe immunodeficiency who have contaminated wounds (including minor wounds) should also receive TIG, regardless of their history of tetanus immunizations.

§ Yes, if ≥10 years since the last tetanus toxoid-containing vaccine dose.

¶ Yes, if ≥5 years since the last tetanus toxoid-containing vaccine dose.
